# Phylogenetic analyses of *Begonia* sect. *Coelocentrum* and allied limestone species of China shed light on the evolution of Sino-Vietnamese karst flora

**DOI:** 10.1186/1999-3110-55-1

**Published:** 2014-01-07

**Authors:** Kuo-Fang Chung, Wai-Chao Leong, Rosario Rivera Rubite, Rimi Repin, Ruth Kiew, Yan Liu, Ching-I Peng

**Affiliations:** 1 School of Forestry and Resource Conservation, National Taiwan University, Daan, Taipei 106 Taiwan; 2 Herbarium (HAST), Biodiversity Research Center, Academia Sinica, Nangang, Taipei 115 Taiwan; 3 Department of Biology, College of Arts and Sciences, University of the Philippines Manila and Philippine National Herbarium, National Museum, Manila, Philippines; 4 Sabah Park, P.O. Box 10626, 88806 Kota Kinabalu, Sabah Malaysia; 5 Forest Research Institute Malaysia, 52109 Kepong, Selangor Malaysia; 6 Guangxi Institute of Botany, Guangxi Zhuangzu Autonomous Region and the Chinese Academy of Sciences, Guilin, 541006 China

**Keywords:** Biological refugia, Cave plants, East Asian monsoon, Phylogenetic niche conservatism, Non-adaptive radiation, Sect. *Diploclinium*, Sect. *Leprosae*, Sect. *Petermannia*, Uplift of Tibetan Plateau

## Abstract

**Background:**

The picturesque limestone karsts across the Sino-Vietnamese border are renowned biodiversity hotspot, distinguished for extremely high endemism of calciphilous plants restricted to caves and cave-like microhabitats that have functioned as biological refugia on the otherwise harsh habitats. To understand evolutionary mechanisms underlying the splendid limestone flora, dated phylogeny is reconstructed for Asian *Begonia*, a species-rich genus on limestone substrates represented by no less than 60 species in southern China, using DNA sequences of nrITS and chloroplast *rpL16* intron. The sampling includes 94 *Begonia* species encompassing most major Asian clades with a special emphasized on Chinese species.

**Results:**

Except for two tuberous deciduous species and a species with upright stems, a majority of Sino-Vietnamese limestone *Begonia* (*SVLB*), including sect. *Coelocentrum* (19 species sampled) and five species of sect. *Diploclinium*, *Leprosae*, and *Petermannia*, are rhizomatous and grouped in a strongly supported and yet internally poorly resolved clade (Clade *SVLB*), suggesting a single evolutionary origin of the adaptation to limestone substrates by rhizomatous species, subsequent species radiation, and a strong tendency to retain their ancestral niche. Divergence-time estimates indicate a late Miocene diversification of Clade *SVLB*, coinciding with the onset of the East Asian monsoon and the period of extensive karstification in the area.

**Conclusions:**

Based on our phylogenetic study, *Begonia* sect. *Coelocentrum* is recircumscribed and expanded to include other members of the Clade *SVLB* (sect. *Diploclinium*: *B. cavaleriei*, *B. pulvinifera*, and *B. wangii*; sect. *Leprosae*: *B. cylindrica* and *B. leprosa*; sect. *Petermannia*: *B. sinofloribunda*). Because species of Clade *SVLB* have strong niche conservatism to retain in their ancestral habitats in cave-like microhabitats and *Begonia* are generally poor dispersers prone to diversify allopatrically, we propose that extensive and continuous karstification of the Sino-Vietnamese limestone region facilitated by the onset of East Asian monsoon since the late Miocene has been the major driving force for species accumulation via geographic isolation in Clade *SVLB*. Morphologically species of Clade *SVLB* differ mainly in vegetative traits without apparent adaptive value, suggesting that limestone *Begonia* radiation is better characterized as non-adaptive, an underappreciated speciation mode crucial for rapid species accumulations in organisms of low vagility and strong niche conservatism.

**Electronic supplementary material:**

The online version of this article (doi:10.1186/1999-3110-55-1) contains supplementary material, which is available to authorized users.

## Background

Karsts are distinctive landforms and hydrological systems formed by the dissolution of highly soluble and porous bedrock (e.g., carbonate rocks such as limestone) with geological ages ranging from the Cambrian to the Quaternary (Ford and Williams^2007^). Around the world, carbonate rocks cover ca. 11% of world’s land surface; however, with the expansion of subterranean hydrological systems, the karst realm makes up more than 14% of the global land areas (Williams^2008^). With more than 800,000 km^2^, Southeast Asia and southern China contain the most extensive limestone karsts on earth (Gillieson^2005^). In particular, the vast karst terrain stretching across the Sino-Vietnamese bordering region (southern China [Guangxi, western Guangdong, southern Guizhou, and southeastern Yunnan] and northern Vietnam; Xu et al.2012a), renowned for spectacular landscapes of fengcongs (cone karst; Waltham^2008^), fenglins (tower karst; Waltham^2008^), and caves, is the largest area on earth with pure carbonate bedrock (Xu^1995^) and has been considered as the model for karst studies (Sweeting^1978^).

Behind these picturesque landscapes, limestone karsts of SE Asia and southern China also abound in rich and marvelous flora with strikingly morphological variation and exceedingly high endemism (Xu^1995^; Clements et al.^2006^; Zhu^2007^). In China, 61 of the ca. 250 Chinese endemic plant genera occur solely in Guangxi (Qin and Liu^2010^), a majority of which are associated with the limestone ecosystem. Of the ca. 470 species (58 genera) of Gesneriaceae recorded from China, at least 200 species (41 genera) are found in Guangxi, with most species confined to limestone karsts. Among them, ca. 100 species and 10 genera are endemic to Guangxi (Qin and Liu^2010^). Of the 18 *Begonia* L. species from Sabah, Borneo associated with limestone substrates, 12 (67%) are only known from a single locality (Kiew^2001^). In Kuching (Sarawak, Boreno), all 15 limestone *Begonia* species are endemic to the local hills only (Kiew^2004^).

Despite their remarkable biodiversity, however, a great proportion of karsts remain to be explored and very little is known regarding the evolutionary processes that have generated the marvelous floras on these limestone landscapes (Clements et al.^2006^). Since *Begonia* is one of the best represented plant groups on tropical limestone karsts (Clements et al.^2006^), it provides an important model for understanding the role that limestone karsts have played in generating high species diversity and endemism.

With recent estimates ranging from ca. 1500 (e.g., Goodall-Copestake et al.^2010^; Thomas et al.2011a) to more than 1600 species (Hoover et al.^2004^), the pantropically distributed *Begonia* sits firmly as one of the ten most species-rich flowering plant genera (Frodin^2004^). However, despite their almost ubiquitous presence in the tropical and subtropical forest ecosystems across Africa, America, and Asia, with few exceptions (e.g., *B. grandis* Dryand., *B. longifolia* Blume, and *B. palmata*; Gu et al.^2007^), most *Begonia* species have very narrow distribution ranges (Tebbitt et al.^2006^; Hughes and Hollingsworth^2008^) and single-site endemic species are common, especially in the limestone karsts (e.g., Kiew^2001^; Peng et al.2008b; Dewitte et al.^2011^).

*Begonia* taxonomy is notoriously challenging because of the genus’ enormous size and often poorly preserved morphological characters in herbarium specimens (Hoover et al.^2004^; Hughes and Girmansyah^2011^), generating great obstacles for further studies. To deal with the unwieldy number of species in the genus, sectional classifications have long been adopted (Doorenbos et al.^1998^; Ku^1999^; Shui et al.^2002^; Kiew^2005^; Gu^2007^). Traditionally these infrageneric classifications have relied primarily on morphological and anatomical characters of fruits and ovaries, with each of the ca. 70 recognized sections confined to one continent only (Doorenbos et al.^1998^), with one known exception (*B. afromigrata* J.J. de Wilde, sect. *Tetraphila*; de Wilde et al.^2011^).

With more than 760 species currently known, Asia harbors the greatest species diversity of *Begonia* (Rajbhandary et al.^2011^; Thomas et al.2011a). In the past decade renewed interest and ongoing explorations to under-collected regions have resulted in the description of more than 140 new species in Asia (e.g., de Wilde et al.^2011^; Thomas et al.2011b; Averyanov and Nguyen^2012^; Peng et al.^2012^,^2013^). Traditionally these Asian species are classified into 22 sections of highly unequal sizes (Doorenbos et al.^1998^; Shui et al.^2002^; Gu^2007^; Hughes and Pullan^2007^; Kiew^2005^; Thomas et al.2011a), with eight of the largest sections: sect. *Coelocentrum* Irmsch., *Diploclinium* (Lindl.) A. DC., *Petermannia* (Klotzsch) A. DC., *Platycentrum* (Klotzsch) A. DC., *Parvibegonia* A. DC., *Reichenheimia* (Klotzsch) A. DC., *Sphenanthera* (Hassk.) Warb., and *Symbegonia* (Warb.) L.L. Forrest & P.M. Hollingsworth, accounting for 95% of the species diversity (Thomas et al.2011a). However, recent molecular phylogenetic analyses have demonstrated the paraphyly (sect. *Platycentrum* and *Petermannia*) or polyphyly (sect. *Diploclinium*, *Leprosae*, *Reichenheimia* and *Sphenanthera*) of most big sections (Tebbitt et al.^2006^; Thomas et al.2011a), indicating the homoplasious or plesiomorphic nature of those morphological characters long emphasized in sectional classification (Tebbitt et al.^2006^; Thomas et al.2011a).

Based on recent treatment of Begoniaceae in the Flora of China (Gu et al.^2007^) and subsequent studies (Liu et al.^2007^; Peng et al.[Bibr CR48][Bibr CR49],[Bibr CR50][Bibr CR52][Bibr CR53]; Li et al.^2008^; Shui^2007^; Wei et al.^2007^), ca. 170 species of *Begonia* assigned to 9 (Shui et al.^2002^) or 7 (Gu^2007^) sections are currently known from continental China, with sect. *Coelocentrum* (47 spp.), *Diploclinium* (41 spp.), and *Platycentrum* (62 spp.) accounting for the majority (88.6%) of the species diversity. Among these, ca. 60 species (35% of the *Begonia* diversity in China) are known primarily from limestone substrates in Guangdong (1 species), Guangxi (44 species), Guizhou (3 species) and Yunnan (19 species) provinces (Gu et al.^2007^; Liu et al.^2007^; Peng et al.[Bibr CR48][Bibr CR49],[Bibr CR50][Bibr CR51][Bibr CR52][Bibr CR53]). These limestone begonias include one species of sect. *Alicida* C.B. Clarke (*B. peii*; Shui et al.^2002^), four species of sect. *Diploclinium* (*B. cavaleriei* H. Lév. [Guangxi, Yunnan, Guizhou], *B. grandis* Dryand. [widespread across eastern China], *B. pulvinifera* C.I Peng & Y. Liu [Guangxi], and *B. wangii*; Gu et al.^2007^), three species of sect. *Platycentrum* (*B. psilophylla* Irmsch. [Yunnan], *B. rubropunctata* S.H. Huang & Y.M. Shui [Yunnan], and *B. subhowii*; Shui et al.^2002^), two species of sect. *Leprosae* (T.C. Ku) Y.M. Shui (*B. cylindrica* D.R. Liang & X.X. Chen [Guangxi] and *B. leprosa* Hance [Guangdong, Guangxi]), one species of sect. *Petermannia* (*B. sinofloribunda*; Shui and Chen^2004^), three species of sect. *Reichenheimia* (*B. chingii* Irmsch. [Guangxi], *B. lithophila* C.Y. Wu [Yunnan], and *B. parvula*; H. Lév. & Vaniot [Yunnan]; Shui et al.^2002^), and all 49 species of sect. *Coelocentrum* (Gu et al.^2007^; Ku et al.^2008^; Liu et al.^2007^; Peng et al.2008a,[Bibr CR50][Bibr CR52][Bibr CR53]).

Morphologically well circumscribed by its parietal placentation and rhizomatous perennation (Shui et al.^2002^; Gu et al.^2007^), *Begonia* sect. *Coelocentrum* is one of the most characteristic limestone plants confined to cave-like microhabitats (i.e., caves, crevices, and fissures) of the Sino-Vietnamese karst region (Peng et al.2008a; Qin and Liu^2010^), with most species known from a single or a few localities and differing from one another by leaf shape, pubescence, texture, and variegation (Gu et al.^2007^). In the past decade, explorations and taxonomic studies in the region have more than tripled the number of species in this section from 15 (Ku^1999^) to over 50 (Gu et al.^2007^;Liu et al.^2007^; Ku et al.^2008^; Peng et al.[Bibr CR48][Bibr CR49],[Bibr CR50][Bibr CR52][Bibr CR53]; Averyanov and Nguyen^2012^;), representing a remarkable example of species radiations across the Sino-Vietnamese limestone karsts comparable to those on oceanic islands and tropical high mountains (Rundell and Price^2009^).

Given the sectional classification schemes, it is tempting to speculate that *Begonia* should have adapted to the limestone substrates at least seven times in China. However, Chinese *Begonia* species, especially those found on limestone substrate, were very poorly sampled in recent phylogenetic analyses (Tebbitt et al.^2006^; Rajbhandary et al.^2011^; Thomas et al.[Bibr CR73][Bibr CR75]). For example, despite its high diversity, only two species of sect. *Coelocentrum* were sampled in the studies by Thomas et al. ([Bibr CR73][Bibr CR75]) and its relationships to other limestone *Begonia* in the region remain to be explored. Additionally, although sect. *Diploclinium* and *Platycentrum*, both major representatives of Chinese *Begonia*, have been proven non-monophyly (Thomas et al.2011a), it remains unclear as to what extent the infrageneric taxonomic framework of Chinese *Begonia* has to be revised, and what mechanisms could have been responsible for the high *Begonia* diversity on the Sino-Vietnamese limestone karsts.

This article studies phylogenetic relationships of limestone *Begonia* of Sino-Vietnamese karsts and concurrently evaluate the monophyly of major *Begonia* sections present in China. To gain further insights into the history of *Begonia* diversification, molecular divergence time estimates are also performed. This study focuses on continental Asian *Begonia* species, complementing recent studies by Thomas et al. ([Bibr CR73][Bibr CR75]) that emphasized the evolutionary patterns of SE Asian *Begonia*.

## Methods

### Taxon sampling

Our sampling strategy aimed to increase the number of species of limestone *Begonia* of continental China not represented in previous molecular phylogenetic analyses, while maximizing representatives of all major clades in Asia (Thomas et al.2011a). In total, 94 Asian species (44 species of mainland China) were sampled, including one species of sect. *Alicida* C.B. Clarke (ALI), eight species of sect. *Baryandra* A. DC. (BAR), 19 species of sect. *Coelocentrum* (COL), 12 species of sect. *Diploclinium* (DIP), one species of sect. *Haagea* (Klotzsch) A. DC. (HAA), four species of sect. *Leprosae* (T.C. Ku) Y.M. Shui (LEP), one species of sect. *Parvibegonia* A. DC. (PAR), 25 species of sect. *Petermannia* (PET), 14 species of sect. *Platycentrum* (PLA), three species of sect. *Reichenheimia* (REI), one species of sect. *Ridleyella* Irmsch. (RID), three species of sect. *Sphenanthera* (SPH), one species of sect. *Symbegonia* (SYM), and *B. boisiana* Gagnep., which is assigned to sect. *Petermannia* by Kiew (2007) but placed as unassignable (UA) to existing section in Doorenbos et al. (1998) and Hughes and Pullan (2007). Two South African species were chosen as outgroups based on previous phylogenetic analyses (Goodall-Copestake et al.^2009^,^2010^; Thomas et al.2011a). Of the 96 species sampled, 49 species (51%) have never been investigated using molecular data. The majority of the sampled species were field-collected plants maintained in the experimental greenhouse of the Academic Sinica, Taipei, Taiwan. Voucher information and GenBank accession numbers are detailed in Additional file1.

### Molecular data

DNA of *Begonia* species is difficult to extract, especially for those collected from limestone substrates. Additionally, silica-gel dried leaves of *Begonia* tend to yield poor quality DNA. To circumvent these issues, total genomic DNA was extracted from fresh leaves of living collections using a modified CTAB protocol optimized for *Begonia* (Kopperud and Einset^1995^).

Two DNA sequence regions were used: the nuclear ribosomal DNA (nrDNA) internal transcribed spacer (ITS) region and the chloroplast DNA *rpL16* intron. Previous analyses based on ITS (e.g., Tebbitt et al.^2006^) suggested its usefulness in resolving interspecific relationships while chloroplast sequences were more suitable for reconstructing the backbone structure of the Asian *Begonia* phylogeny (Thomas et al.2011a). Although the *rpL16* intron has thus far never been used in reconstructing the *Begonia* phylogeny, this region amplified and sequenced easily and shows adequate phylogenetic resolution in Asian *Begonia*. For each polymerase chain reaction (PCR), amplification was performed in a total volume of 25 μl, including 12.5 μl of *Taq* DNA Polymerase Master Mix Red (Ampliqon, Copenhagen, Denmark), 1 μl of each forward and reverse primer (10 μM), 2 μl of template DNA, and 8.5 μl of ddH_2_O. For ITS, the primers 5P and 26S1Rev primers (Clement et al.^2004^) were used for both PCR amplification and sequencing. For *rpL16* intron, three primers (rpL16-F: GCT ATG CTT AGT GTG TGA CTC G; rpL16-R: CGT CCY GCT TCT ATT TGT CTA G; Beg_rpL16: GTT TCA CAT TAT CTG GAT CG) were designed to optimize PCR amplification (rpL16-F and rpL16-R) and sequencing (Beg_rpL16 and rpL16-R) in *Begonia*. PCR reactions were carried out by a denaturation-step in 94°C for 5 min, 30 thermo-cycles of 94°C for 30 s, 60°C for 30 s, and 72°C for 90 s (60 s for ITS), and a final extension in 72°C for 5 min. PCR products were purified using QIAquick PCR purification Kit (Qiagen, Valencia, California, U.S.A.) and then sequenced using an ABI PRISM dye terminator cycle sequencer, model 3700 (Applied Biosystems, Foster City, California, U.S.A.).

### Phylogenetic analyses

DNA sequences were aligned using the program MUSCLE implemented in the software MEGA5 (Tamura et al.^2011^) with subsequent manual adjustments. The aligned matrix was analyzed using maximum parsimony (MP) and maximum likelihood (ML) optimality criteria and Bayesian Inference (BI). MP analyses were performed using PAUP* v.4.0b10 (Swofford^2002^). All characters were unordered and equally weighted and gaps were treated as missing. Heuristic searches were conducted with 1000 replicates of random addition, 10 trees held at each step during stepwise addition, tree-bisection-reconnection (TBR) branch-swapping, and deepest descent option in effect. Brach supports (parsimony bootstrap: PB) were assessed by 1000 bootstrap replicates with 10 random taxon additions and heuristic search options. To assess the congruence among the two data sets, the incongruence length difference test (ILD test for partition-homogeneity test in PAUP*) was performed. One thousand homogeneity replicates of heuristic searches were conducted with 100 random sequence additions and MulTrees not in effect. Ten ML analyses with 5000 bootstrap (likelihood bootstrap: LB) resamplings were conducted using RAxML-HPC2 v7.2.7-3 (Stamatakis et al.^2008^) via the CIPRES Portals (http://www.phylo.org/index.php/portal/). The matrix was partitioned (i.e., ITS vs. *rpL16* intron) with a gamma model of rate heterogeneity and proportion of invariable sites estimated by the program. The program MrBayes v3.1.2 (Ronquist and Huelsenbeck^2003^) was employed for BI analyses. The aligned matrix was partitioned into ITS and *rpL16* data sets. Prior to the analysis, DNA substitution models for each partition were selected by MrModeltest (Nylander^2004^) based on the Akaike information Criterion (AIC). BI analyses were conducted using mixed models specified to each data partition. Four runs of Metropolis-coupled Markov chain Monte Carlo (MCMCMC) analyses were performed, with a random starting tree and four chains for each run (one cold and three heated). The MCMCMC length was ten million generations and the chain was sampled every 1000th generation from the cold chain. To assure that the cold chain swapped successfully with the heated chains and the Metropolis coupling functioned well, the resulting log files were by inspecting the trace plots of parameters using the program Tracer v1.5 (Drummond and Rambaut^2007^). The function ‘Compare and Cumulative’ of the program AWTY (Nylander et al.^2008^) was employed to check topological convergence and convergence of posterior probabilities (PP) of splits from different runs. Bayesian clade posterior probabilities and average branch lengths were calculated based on the sampled trees after the first 25% of the sampled trees were discarded as burn-in.

### Molecular dating

**Figure 1:**
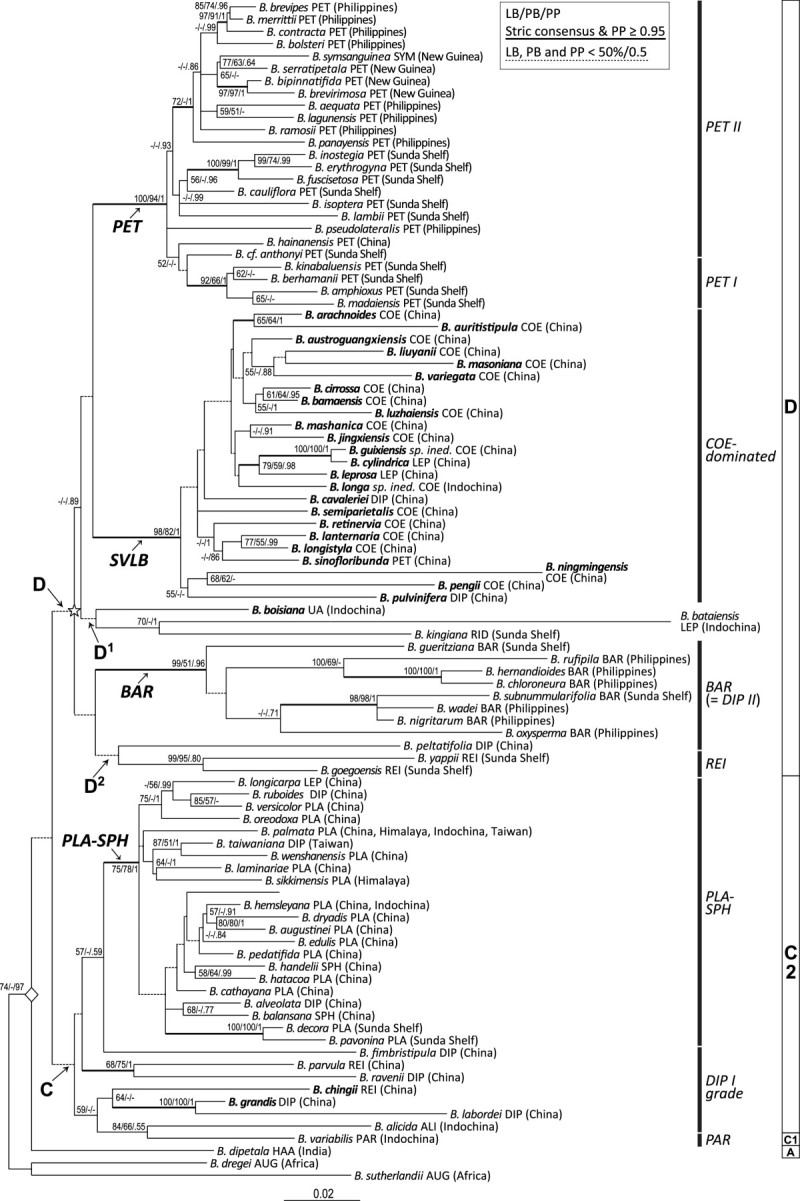
**Best-scoring maximum likelihood phylogram.** Clade support values (LB: likelihood bootstrap/PB: parsimony bootstrap/PP: posterior probability) larger than 50% are indicated at each node. Dashed branches indicate LB, PB, and PP all smaller than 50%/0.5 while thick branches denote those present in the strict consensus tree of MP analysis and PP ≥0.95. Species in bold denote limestone species distributed in the Sino-Vietnamese limestone karsts (Gu et al.,^2007^). Arrows point to clades and sections discussed in the text. Taxon names are followed by sectional placement and distribution (in parentheses). The rhombus (◇) and star (☆) signs denote calibration points for molecular dating. Sectional classification and clade names in Thomas et al. (2011a) are marked to the right to allow easier cross-study comparison. Sources of sectional placement for each species are cited in Additional file1. Sectional abbreviations: ALI: *Alicida*, AUG: *Augustia*, BAR: *Baryandra*, COL: *Coelocentrum*, DIP: *Diploclinium*, HAA: *Haagea*, LEP: *Leprosae*, PAR: *Parvibegonia*, PET: *Petermannia*, PLA: *Platycentrum*, REI: *Reichenheimia*, RID: *Ridleyella*, SPH: *Sphenanthera*, SYM: *Symbegonia*, UA: unassigned.

The software package BEAST v1.6.1 (Drummond and Rambaut^2007^) was employed to perform divergence-time estimates. The aligned matrix was formatted for BEAST using BEAUti with the data partitioned to allow specification of DNA substitution models for each partition (see BI analysis above). Because suitable fossils in Begoniaceae are not available, the phylogenetic tree was calibrated using two normally distributed priors based on divergence time estimated by Thomas et al. (2012) for Asian *Begonia*: the mean of estimated divergence ages of the crown node of Asian *Begonia* (Clade 10 in Figure three of Thomas et al.^2012^; the node marked with a rhombus sign in Figure 1) at 16.1 (95% Highest Posterior Density range [HPD] 9.1–22.5) mya (million years ago) and Clade 49 in Figure three of Thomas et al.^2012^ (clade D, marked with a star sign in Figure 1) at 13.0 (7.7–18.9 HPD) mya. Clade D in Figure three of Thomas et al. (2012) includes sect. *Coelocentrum* and *Petermannia*, and species of sect. *Diploclinium* (Clade *BAR*) centered in the Philippines recently classified under the recircumscribed sect. *Baryandara* (Rubite et al.^2013^). For these two clades, the calibration priors were modeled with the mean of 16.1 mya and the standard deviation of 3.265 (95% probability interval: 9.7–22.5 mya) for the Asian *Begonia* Clade and the mean of 13.0 mya and the standard deviation of 2.7 (95% probability interval: 7.7–18.3 mya) for Clade 49 to account for HPDs of age estimates of the original analysis. The tree prior was set to the Yule process and a single uncorrelated-rates relaxed molecular clock model assuming a lognormal distribution of rates (UCLD) was applied to all partitions. Two independent runs of BEAST analyses were conducted, each with 50 million generations and sampling every 1,000th generation.

## Results

Information of sequence variability in each partition is summarized in Table 1. The alignment file is available upon request to the corresponding author (CIP). The combined data matrix included 1974 aligned positions (ITS: 864 bp; *rpL16*: 1110 bp), of which 29% of the aligned sequences (31.6% in ITS and 26.9% in *rpL* 16) were excluded because of uncertain alignment. MP analyses yielded 149503 equally parsimonious trees of 3480 steps (CI = 0.4086; RI = 0.5542; RC = 0.2264). For BI analyses, AIC selected the model GTR+G+I for ITS and GTR+G for *rpL16*.

**Table 1:** **Summary of sequence variability**

Partition	Length variation (bp)	No. of aligned position (bp)	Variable characters (%)	Parsimony informative character (%)	No. of characters excluded (#%)
ITS	504–730	864	591 (68.4%)	405 (46.9%)	273 (31.6%)
*rpL16*	797–973	1110	336 (30.3%)	123 (11.1%)	299 (26.9%)
Combined	1440–1658	1974	927 (50.0%)	528 (26.7%)	572 (29.0%)

The result of ILD test (*P* = 0.001) indicates significant incongruence between ITS and the *rpL16* data sets. However, because ILD test has been doubted for generating misleading conclusions under certain circumstances (Yoder et al.^2001^), topologies of MP analysis of ITS, *rpL16*, and the concatenated data sets were compared (Wiens^1998^). While relationships generated from ITS and the combined data sets are highly congruent, topology of *rpL16* is largely incongruent with the formers (data not shown). Nevertheless, supports for the conflict relationships between ITS (and the concatenated) and *rpL16* topologies are all less than 50% and the combined data set has much higher support values for most clades, suggesting a ‘soft incongruence’ (Wiens^1998^). Therefore, the two data sets were combined for all subsequent analyses.

For MP and ML analyses, 70–79%, 80–90%, and 90–100% bootstrap supports (LB and PB) are considered as moderate, well, and strongly supported relationships, respectively. For BI analyses, the threshold for a well-supported clade is 95% PP. The best scored ML tree (ML optimization likelihood = -20967.197060), with bootstrap support values (PB and LB) and the posterior clade probabilities (PP), is depicted in Figure 1. Clades designated in Thomas et al. (2011a) and sectional classification are also marked to the right of the ML tree in Figure 1 to allow easier cross-comparisons between studies.

Topology of the best-scoring ML tree is largely compatible with those uncovered in recent phylogenetic analyses of similar scale (Rajbhandary et al.^2011^; Thomas et al.2011a), though relationships among deeper nodes are very poorly supported (Figure 1). In the best-scoring ML tree, all sampled Asian *Begonia* species form a moderately supported clade (LB: 74) with strong support in BI analysis (PP: 0.97). Within the Asian Clade, the Indian species *B. dipetala* Graham (sect. *Haagea*) is sister to a clade composed of all the remaining Asian species, though lacking support (Figure 1). Within this latter clade, species are grouped with low supports into two clades (Clades C and D; Figure 1) corresponding to Clade C1 + C2 and Clade D in Thomas et al. (2011a).

Within Clade C, eight sampled species of tuberous and deciduous habit form a poorly-supported successive grade (Figure 1) sister to a moderately to strongly supported Clade *PLA-SPH* (LB: 75, PB: 78, PP: 1). This grade, corresponding well (except for sect. *Parvibegonia*) to the *DIP I* grade in Thomas et al. (2011a), includes two Indochinese species (sect. *Alicida*: *B. alicida*; sect. *Parvibegonia*: *B. variabilis*), five Chinese species (sect. *Diploclinium*: *B. fimbristiupla*, *B. grnadis*, and *B. labordei*; sect. *Reichenheimia*: *B. chingii* and *B. parvula*), and one species of Taiwan (sect. *Diploclinium*: *B. ravenii* C.I Peng & Y.K. Chen). Clade *PLA-SPH* is mainly composed of species of sect. *Platycentrum* and *Sphenanthera*, plus one species of sect. *Leprosae* (*B. longicarpa* K.Y. Guan & D.K. Tian) and two species of sect. *Diploclinium* (*B. alveolata* T.T. Yu and *Begonia ruboides* C.M. Hu ex C.Y. Wu & T.C. Ku). Of the Chinese species of the *DIP I* grade, *B. chingii* and *B. parvula* are distributed exclusively on limestone substrates of Guangxi and Yunnan, respectively, while *B. grandis* is the most widespread *Begonia* species in China found in a variety of habitats including limestone (Gu et al.^2007^).

The poorly resolved Clade D (Figure 1) comprises five subclades: two poorly supported (Clades D^1^ and D^2^) and three strongly (Clades *PET*, *SVLB*, and *BAR*). Clade D^1^ consists of three species restricted to limestone outcrops: *B. bataiensis* Kiew (sect. *Leprosae*) from southwestern Vietnam (Tam et al.^2005^), *B. kingiana* (sect. *Ridleyella*) from Peninsular Malaysia (Kiew^2005^), and *B. boisiana* from central and northern Vietnam (Tebbitt^2005^) of the southern limit of the Sino-Vietnamese limestone karsts. Clade D^2^ is composed of two species of sect. *Reichenheimia* of Sunda Shelf (*B. goegoensis* N.E. Br. and *B. yappii* Ridl.) and *B. peltatifolia* H.L. Li (sect. *Diploclinium*) endemic to Hainan, China (Gu et al.^2007^). This latter species is morphologically assignable to the redefined sect. *Baryandra* (= Clade *BAR* in Figure 1 or *DIP II* in Thomas et al.2011a), a strongly supported clade expanding Philippines’ monophyletic sect. *Baryandra* (*B. oxysperma*) to include species of sect. *Diploclinum* centered in the Philippines and adjacent SE Asian islands (Rubite^2012^; Rubite et al.^2013^). The strongly supported (LB: 100, PB: 94, PP: 1) Clade *PET* is composed of all sampled species of the species-rich SE Asian sect. *Petermannia* (including sect. *Symbegonia*) except for *B. sinofloribunda*, which is nested within Clade *SVLB* with strong supports (LB: 98, PB: 82, PP: 1). With the addition of *B. sinofloribunda*, a rhizomatous species distributed in southern Guangxi, the clade of Sino-Vietnamese limestone *Begonia* (Clade *SVLB*) encompasses all sampled rhizomatous limestone *Begonia* distributed in southern China and northern Vietnam, including all sampled species of sect. *Coelocentrum*, two species of sect. *Leprosae* (*B. leprosa* Hance and *B. cylindrica* D.R. Liang & X.X. Chen), and two species of sect. *Diploclinium* (*B. cavaleriei* H. Lév. and *B. pulvinifera* C.I Peng & Yan Liu). Within the strongly supported Clade *SVLB*, phylogenetic resolutions are low and weakly supported (Figure 1).

**Figure 2:**
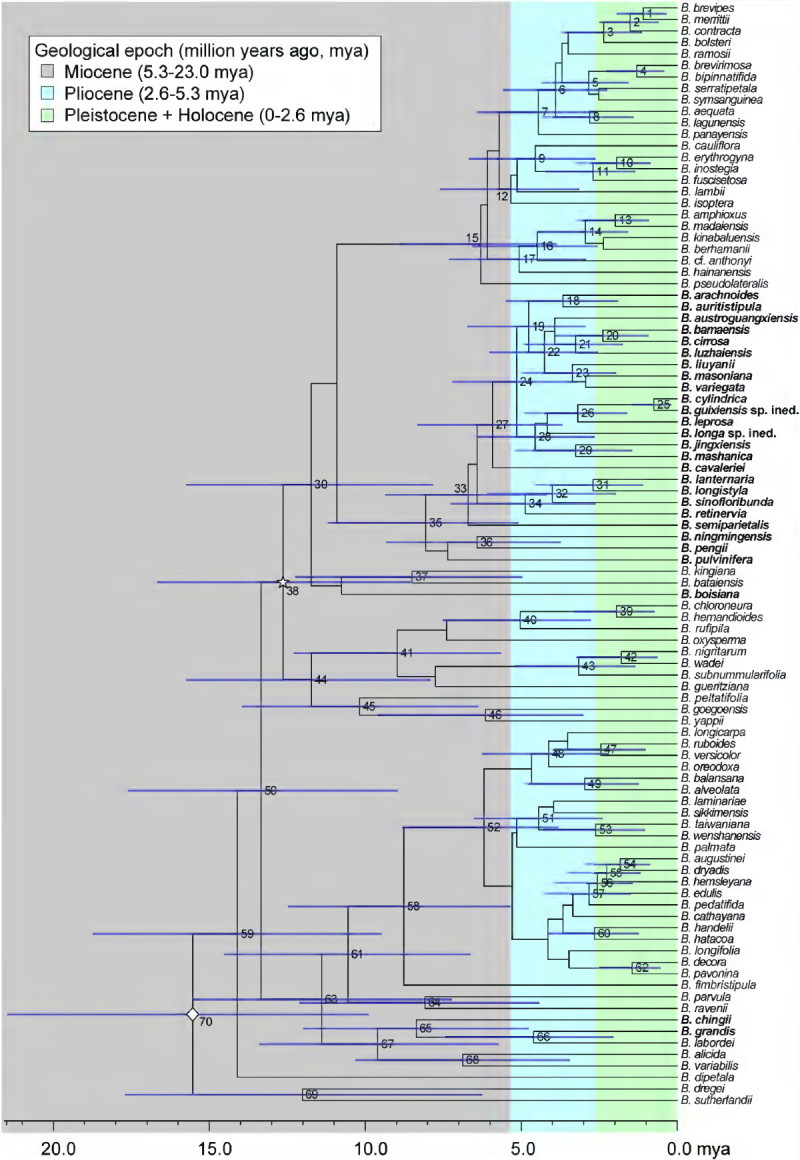
**Maximum clade credibility chronogram estimated by BEAST.** The rhombus (◇) and star (☆) signs denote calibration points for molecular dating. Dashed clades indicate PP < 0.75 and thicken clades indicate PP ≥ 0.95. Node heights indicate mean ages, with 95% highest posterior density (HPD) date ranges shown by the node bars. Clades with PP ≥ 0.75 are numbered and their clade support and divergent ages mean (95% HPD) are shown in Table 2.

The maximum clade credibility chronogram and the summary of the divergence time estimates and clade supports (PP) generated by the BEAST software are shown in Figure 2 and Table 2

**Table 2:** **Clade supports and divergent age estimates for Asian** ***Begonia***

Clade	Clade support (PP)	Divergent ages mean (95% HPD) mya
1	0.96	1.09 (0.34–1.96)
2	1.00	1.51 (0.60–2.50)
3	1.00	2.36 (1.12–3.71)
4	1.00	1.30 (0.42–2.28)
5	0.90	2.84 (1.55–4.34)
6	0.83	3.90 (2.25–5.59)
7	1.00	4.45 (2.58–6.42)
8	0.96	2.80 (1.39–4.32)
9	0.94	4.55 (2.61–6.69)
10	1.00	1.94 (0.85–3.22)
11	1.00	2.7 (1.35–4.23)
12	0.97	5.33 (3.14–7.61)
13	1.00	1.98 (0.91–3.2)
14	1.00	2.94 (1.57–4.48)
15	1.00	6.30 (3.87–8.89)
16	0.92	4.48 (2.56–6.57)
17	1.00	5.07 (2.92–7.32)
18	1.00	3.66 (1.88–5.50)
19	0.76	4.76 (2.94–6.73)
20	0.84	2.39 (0.91–3.92)
21	0.99	3.25 (1.73–4.95)
22	0.99	4.26 (2.54–6.03)
23	1.00	3.36 (1.96–4.96)
24	0.93	5.15 (3.21–7.21)
25	1.00	0.76 (0.19–1.46)
26	1.00	3.18 (1.60–4.9)
27	0.78	5.91 (3.67–8.34)
28	0.77	4.55 (2.66–6.43)
29	1.00	3.25 (1.44–5.19)
30	0.83	11.74 (7.83–15.75)
31	1.00	2.7 (1.09–4.53)
32	0.91	4.01 (1.97–6.10)
33	0.99	6.7 (4.18–9.36)
34	1.00	4.87 (2.61–7.26)
35	1.00	8.06 (5.09–11.21)
36	0.84	6.42 (3.74–9.33)
37	0.99	8.5 (4.97–12.25)
38*	0.80	12.64 (8.54–16.67)
39	1.00	1.95 (0.72–3.32)
40	1.00	5.03 (2.76–7.53)
41	1.00	8.98 (5.64–12.30)
42	0.95	1.80 (0.63–3.24)
43	1.00	3.16 (1.35–5.21)
44	0.81	11.73 (7.90–15.75)
45	0.80	10.18 (6.38–13.95)
46	1.00	6.15 (3.00–9.59)
47	0.99	2.45 (1.01–3.99)
48	0.96	4.12 (2.18–6.27)
49	1.00	2.97 (1.23–4.87)
50	0.83	13.34 (8.96–17.61)
51	1.00	4.44 (2.39–6.51)
52	1.00	6.20 (3.80–8.82)
53	1.00	2.61 (1.04–4.29)
54	0.98	1.83 (0.86–2.92)
55	0.94	2.27 (1.18–3.50)
56	0.77	2.57 (1.41–3.90)
57	0.98	2.83 (1.50–4.21)
58	0.98	8.76 (5.34–12.49)
59	1.00	14.1 (9.45–18.71)
60	0.92	2.65 (1.23–4.14)
61	0.75	10.55 (6.63–14.52)
62	1.00	1.44 (0.53–2.50)
63	0.92	11.4 (7.23–15.52)
64	0.99	8.08 (4.41–12.11)
65	0.90	8.36 (4.76–12.00)
66	1.00	4.6 (2.03–7.44)
67	1.00	9.6 (5.73–13.41)
68	1.00	6.88 (3.44–10.31)
69	0.81	12.02 (6.25–17.71)
70*	1.00	16.1 (9.89–21.48)

Asterisks indicate calibration points.

The crown age of Clade 38, corresponding to Clade D of Thomas et al. (2011a) and Clade D49 of Thomas et al. (2012) that includes sect. *Coelocentrum*, *Petermannia,* and *Baryandra* (= *DIP II*), was estimated to be 12.64 mya, with the HPD date ranging from 8.54 to 16.67 mya. For Clade *SVLB* the mean divergent crown age was estimated to be 8.06 mya, with the HPD date ranging from 5.09 to 11.21 mya (Figure 2; Table 2).

## Discussion

Although relationships among deep nodes of Asian *Begonia* remain poorly supported in our current study, the basic topology and major lineages identified (Figure 1) are largely congruent with previous studies (Tebbitt et al.^2006^; Rajbhandary et al.^2011^; Thomas et al.[Bibr CR73][Bibr CR75]) with some exceptions. For instance, our ML tree (Figure 1) indicates a sister relationship between the Indian species *B. dipetala* (sect. *Haagea*) and the rest of Asian *Begonia*, while *B. dipetala* was placed sister to Clades B + C1 + C2 with weak support in Thomas et al. (2011a). With the exclusion of *B. sinofloribunda*, sect. *Petermannia* (Clade *PET*) of Clade D is monophyletic with strong support (Figure 1); however, in Thomas et al. (2011a) this section is polyphyletic, separated into two strongly clades (Clade *PET I* and *PET II*) with Clade *PET I* basal to the subclade encompassing Clades *PET II*, *BRA* (sect. *Bracteibegonia* A. DC.), *DIP II* (= Clade *BAR* in Figure 1), *REI*, and *B. kingiana* (sect. *Ridleyella*). It is worth to mention, however, that Clade *BAR* is the basal clade of the *Platycentrum*-*Sphanenthera* clade in Tebbitt et al. (2006). Additionally while Clade *SVLB* is placed sister to sect. *Petermannia* in present study (Figure 1), sect. *Coelocentrum* is the basal clade sister to the rest of Clade D in Thomas et al. (2011a). However, except for the monophyly of Clade *PET* (*PET I* + *PET II*) in present study, supports for relationships of these deep nodes are low, indicating that they are mostly soft incongruence (Wiens^1998^). Additionally conflicts between our and previous studies could also be attributable to the limited phylogenetic signals of the molecular markers employed in the current analyses. Alternatively, past hybridization events could have also led to the incongruence between the chloroplast-based relationships in Thomas et al. (2011a) and those unveiled by our study using the combination of ITS and chloroplast markers (Goodall-Copestake et al.^2009^; Dewitte et al.^2011^; Thomas et al.2011a), as reports on interspecific hybridizations have become increasingly common in Asian *Begonia* (e.g., Peng et al.^2010^). Because a full discussion of phylogenetic relationships of Asian *Begonia* is beyond the scope of present study, our discussion is limited to well-supported clades pertaining to the evolution of limestone *Begonia* of China.

### Taxonomic implications of the phylogenetic analyses

Based on a very different set of taxonomic sampling, our analyses corroborate results of previous analyses (Tebbitt et al.^2006^; Thomas et al.2011a) in confirming the non-monophyly of most major Asian sections, including sect. *Diploclinium*, *Leprosae*, *Petermannia, Platycentrum*, *Reichenheimia*, and *Sphenanthera* (Figure 1). Additionally, our expanded sampling of Chinese *Begonia* species further demonstrates the paraphyly of sect. *Coelocentrum*, dominating in the strongly supported Clade *SVLB* otherwise also composed of two species of sect. *Diploclinium* (*B. cavaleriei* and *B. pulvinifera*), two species of sect. *Leprosae* (i.e., *B. cylindrica* and *B. leprosa*), and one species of sect. *Petermannia* (*B. sinofloribunda*). Despite being one of the biggest (47 species) and morphologically most diverse sections in China, only three rhizomatous species of sect. *Diploclinium* (*B. cavalerieri*, *B. pulvinifera*, and *B. wangii*) are known from the Sino-Vietnamese limestone karsts (Gu et al.^2007^; Peng et al.^2006^), none sampled in previous phylogenetic analyses. Our study thus further strengthens the polyphyly of sect. *Diploclinium* and the homoplasious nature of its diagnostic characters, i.e., bilamellate, 3-locular axillary placentation, in Asian *Begonia* (Thomas et al.2011a).

Sect. *Leprosae*, raised for three species (*B. cylindrica*, *B. leprosa* and *B. longicarpa*) bearing cylindric and berry-like fruits in southern China (Shui et al.^2002^), has been questioned to be artificial when *B. bataiensis* Kiew, a tuberous species bearing the characteristic fruit type from limestone karsts of southwestern corner of Vietnam described (Tam et al.^2005^). Subsequently molecular study sampling two species (*B. leprosa* and *B. longicarpa*) showed that sect. *Leprosae* is polyphyletic with its type species *B. leprosa* (limestone species) placed sister to sect. *Coelocentrum* and *B. longicarpa* (not limestone species) nested within clade dominated by sect. *Platycentrum* and *Sphenanthera*, suggesting that the cylindric and berry-like fruits evolved independently (Tebbitt et al.^2006^). With all four recognized species sampled, our analyses attested further the polyphyly of sect. *Leprosae*, with two limestone species (*B. cylindrica* and *B. leprosa*) in the Sino-Vietnamese karsts nested within Clade *SVLB*, *B. longicarpa* in Clade *PLA-SPH*, and *B. bataienssi* sister to *B. kingiana* (Figure 1).

Known only from hills of limestone karsts of the southern Guangxi adjacent to the Vietnamese border (Gu et al.^2007^), *B. sinofloribunda*, a rare species uniquely characterized by branched aerial, subwoody stems and pendulous inflorescences with small flowers and ovaries showing axillary placentas, has been variously classified in sect. *Platycentrum* (Gu^2007^; Ku^1999^), *Diploclinium* (Shui et al.^2002^ and *Petermannia* (Shui and Chen^2004^). Our analysis nevertheless indicates strongly that *B. sinofloribunda* is derived from within Clade *SVLB* (Figure 1), greatly increase the morphological diversity of Clade *SVLB*.

Based on our study, although species of Clade *SVLB* vary greatly in their ovary anatomy and fruit type that are crucial for sectional classification in *Begonia*, they are all rhizomatous distributed exclusively in cave-like microhabitats of the limestone karsts of the Sino-Vietnamese bordering areas. Additionally all available data, including an unpublished chromosome count of *B. sinofloribunda* (Peng, unpublished data), indicate that members of Clade *SVLB* are uniformly characterized by the chromosomal number 2*n* = 30 Gu et al. (2007; Peng et al.^2010^; Thomas et al.2011a) or its triploid number 2*n* = 45 (Peng et al.^2013^). Given the obvious cohesiveness in their perennation organs as well as ecology, biogeography and chromosome cytology, we thus propose to expand the definition of sect. *Coelocentrum* (see Taxonomic Treatment) to include *B. sinofloribunda*, three species of sect. *Diploclinium* (*B. cavaleriei, B. pulvinifera*, and *B. wangii*; see Taxonomic Treatment) and two species of sect. *Leprosae* (*B. cylindrica* and *B. leprosa*).

### Phylogenetics and molecular dating of Clade *SVLB*

Except for species of Clade *SVLB*, the only *Begonia* species found on the Sino-Vietnamese limestone karsts are the tuberous *B. chingii* Irmsch. and *B. grandis* (Gu et al.^2007^) of the *DIP I* grade (Figure 1) and *B. boisiana* that possesses upright stems (Tebbitt^2005^). The phylogenetic relationships therefore indicate that, in the Sino-Vietnamese limestone karsts, *Begonia* had evolved to adapt to the harsh limestone habitats four times, twice evolved with the tuberous and deciduous habit (or once and secondary invading the none-limestone habitat by *B. labordei*, Figure 1), once by the species with upright stem (*B. boisiana*), and once by the rhizomatous ancestor of Clade *SVLB* (Figure 1). Interestingly, although tubers and a deciduous habit appear to be suitable for surviving in the desiccation-prone limestone habitats such as most karst *Begonia* in Madagascar (Grubb^2003^; Keraudren-Aymonin^1983^), our data strongly suggest that it is the rhizomatous Clade *SVLB* that succeeded and proliferated in the Sino-Vietnamese limestone karst terrains.

Within the strongly supported Clade *SVLB*, phylogenetic resolution is poor (Figure 1), possibly indicating a rapid species radiation (Hughes and Eastwood^2006^; Kozak et al.^2006^). Based on the divergence times calculated by BEAST, the crown age of Clade *SVLB* (Clade 35 in Figure 2; Table 2) was estimated to be ca. 8.06 (HPD 5.09–11.21) mya during the middle to late Miocene, with the stem age extending back to ca. 11 mya (Figure 2). Although these age estimates are likely problematic because of the employment of secondary calibration, even within the lowest end (ca. 5 mya), the crown age of the Sino-Vietnamese limestone *Begonia* is much older than those estimated in recent phylogenetic literature such as species radiations triggered by the Pleistocene uplift of the South American Andes (e.g., Hughes and Eastwood^2006^; Hoorn et al.^2010^).

Interestingly, the estimated crown age of Clade *SVLB* at 8.06 (HPD 5.09–11.21) mya (Table 2; Figure 2) appears to coincide well with the most extensive stage of karstifications in Guangxi during the Miocene (Liu^1997^,^2003^) and the onset and intensification of the East Asian monsoon at ca. 7.2 (An^2000^) to 11 mya (Zheng et al.^2004^). In Asia, the climate has been affected strongly by the altitude of the Himalayas and the Tibetan Plateau. By the late Miocene, the coeval uplift of Himalaya-Tibetan region had attained significant heights that greatly altered Asian atmospheric circulation, resulting in the onset of the warm and humid East Asian summer monsoon (An et al.^2001^). Because high temperature and precipitation brought by the summer monsoon are favorable conditions for the development of karsts (Zhang^1989^), the late Miocene onset of the East Asian monsoon would have greatly facilitated the development of limestone karsts across the Sino-Vietnamese limestone regions (Liu^1997^,^2003^), resulting in the remarkable landscapes and providing ample habitats for the spreading and diversification of limestone karst plants such as *Begonia*. Recently the uplift of Himalaya-Tibetan region and intensification of East Asian monsoon have also been suggested to be responsible for triggering the rapid diversification of both seasonally and wet adapted *Begonia* in continental Asia (Rajbhandary et al.^2011^) and the fern genus *Lepisorus* in China and Japan (Wang et al.^2012^).

### Diversity pattern of Clade *SVLB*

Species of the Clade *SVLB*, comprising ca. 60 species (see Taxonomic Treatment) mainly distributed in Guangxi, inhabit the ‘twilight zone’ (Poulson and White^1969^) of limestone caves and cave-like microhabitats such as shaded fissures and crevices abundantly occurring throughout the Sino-Vietnamese limestone karst region (Sweeting^1978^; Schindler^1982^; Xu^1995^; Zhu^2007^). Such cave-like microhabitats, characterized by constant temperature, high humidity, and indirect and low light (Liang et al.^2010^), function as shelters on the limestone bedrock characterized by highly alkaline conditions, thin soil layers, and severe desiccation due to high porosity (Clements et al.^2006^). In addition to *Begonia*, these cave-like microhabitats are also inhabited by several species-rich, calciphilous herbaceous plant groups such as *Aspidistra* Ker Gawl. (e.g., Liu et al.^2011^), a number of genera of gesnerids (e.g., Weber et al.^2011^; Xu et al.[Bibr CR86][Bibr CR87]), *Elatostema* L. (e.g., Wei et al.^2011^), *Impatiens* (e.g., Yu et al.^2009^), and the fern genus *Polystichum* (e.g., Zhang and He^2011^), etc. Many of these limestone plants are often highly ornamental and have long been commercially exploited (Vermeulen and Whitten^1999^). Biogeographically most of these calciphilous cave plant species have a very limited distribution ranges and site-endemics are very common (Liu et al.^2007^; Peng et al.[Bibr CR49][Bibr CR52]; Xu et al.2012a; Zhang and He^2011^). In individual caves or crevices, species diversity is often low; however, across the landscapes, plant composition varies greatly from one cave to another, resulting in low α diversity and yet extremely high β diversity across the landscape (Hughes and Hollingsworth^2008^).

### Phylogenetic niche conservatism of Clade *SVLB*

The monophyly of the rhizomatous Clade *SVLB* suggests a single evolutionary origin of the adaptation to the cave-like limestone microhabitats, and shows that ecology is good indicator of taxonomic relationships than traditional morphological characters used to defined sections. Inspecting the chronogram (Figure 2) reveals a 3 MY-time lag between the estimated stem and crown age of Clade *SVLB*, which might signify a long period needed for their ancestor to gradually adapt to the calcium-rich and cave-like microhabitats on the limestone karsts before the commencement of species radiation. Its monophyly and prolonged existence since the late Miocene also suggest the strong tendency in the evolution of this clade to retain the ancestral ecological niche, presenting an apparent case of strong *‘phylogenetic niche conservatism’* (Kozak et al.^2006^; Wiens^2004^).

### Cave-like microhabitat as paleorefugia

Limestone caves and crevices/fissures are formed through the development of underground hydrological systems with maximum discharge and dissolutional aggressiveness (Palmer^1991^). The development and ‘collapse’ of caves are essential processes shaping the karst topography (Ford and Williams^2007^; Palmer^1991^). In more recent histories, anthropogenic factors have also greatly accelerated the destruction of caves and cave-like microhabitats in the karsts (Clements et al.^2006^). Together with the observations of the unique ecology of limestone *Begonia* ,limestone caves and crevices currently inhabited by rich plant communities can be viewed as “*biological refugia, habitats that support populations not able to live elsewhere in a landscape* (Nekola^1999^”. More precisely, these cave-like microhabitats in the Sino-Vietnamese limestone karsts qualify as “*paleorefugia, now-fragmented relicts of a formerly widespread matrix community* (Nekola^1999^)”. On the contrary, “*neorefugia represent habitats that are younger than the surrounding biological matrix* (Nekola^1999^)”.

### Clade *SVLB* as an example of a non-adaptive radiation on limestone karsts

The onset of Asian summer monsoon in the late Miocene (An et al.^2001^) provided ideal conditions for the karst development (Zhang^1989^) across the limestone terrains of the Sino-Vietnamese bordering region (Liu^1997^), resulting in numerous crevices, fissures, and caves and the splendid fenglins and fengcongs across the landscapes. Although limestone karsts are an unsuitable growth substrate for most plants, over time, some plant groups (e.g., *Aspidistra*, *Begonia*, *Impatiens,* various gesnerid genera, *Elatostema*, etc.) adapted to and flourishing in the cave-like microhabitats. However, cave-like microhabitats are fragile and collapsed constantly due to the processes of karstification (Palmer^1991^) associated with the persistence of East Asian monsoon (An et al.^2001^; Liu^1997^), resulting in fragmentation and isolation of these microhabitats. Moreover, because of the strong niche conservatism (Wiens^2004^) to the cave-like microhabitats, populations of the calciphilous plants had been forced to track the paleorefugia (Wiens^2004^; Kozak et al.^2006^; Comes et al.^2008^). Moreover, because *Begonia* species are poor dispersers (Hughes and Hollingsworth^2008^), gene flow among populations would be retarded, greatly augmenting effects of random genetic drift (Comes et al.^2008^; Hughes and Hollingsworth^2008^). As geological weathering processes have occurred continuously throughout the Sino-Vietnamese limestone karsts, population diversification likely would have resulted in steady accumulation of species (Kozak et al.^2006^; Comes et al.^2008^; Hughes and Hollingsworth^2008^). However, because the major driving force of this evolutionary process is geographic isolation, the diversity pattern is better characterized as ‘non-adaptive radiation’, an underappreciated speciation mode crucial for rapid species accumulations in organisms of low vagility and strong niche conservatism (Gittenberger^1991^; Kozak et al.^2006^; Comes et al.^2008^; Rundell and Price^2009^). Morphologically, species of Clade *SVLB* all have very similar flowers, distinguishing from one another mainly by vegetative characters such as leaf morphology in shape, pubescence, texture, and variegation (Gu et al.^2007^). Recent studies, however, show that different variegations in *Begonia* leaves have no apparent adaptive differences (Sheue et al.^2012^; Zhang et al.^2009^), suggesting that the species accumulation in *Begonia* was mainly a non-adaptive process (Gittenberger^1991^; Kozak et al.^2006^; Comes et al.^2008^; Rundell and Price^2009^), though further studies are definitely needed to test the non-adaptive nature of leaf morphology in Clade *SVLB*.

## Conclusions

The proposed scenario of species diversification in Clade *SVLB* driven by geological and climatic conditions in the Sino-Vietnamese limestone karst should have also affected other calciphilous cave plants in the region, resulting in concordant phylogenetic patterns across lineages. It is also worth noting that the biology of those calciphilous limestone karst plants appears to be highly similar to some amphibians (e.g., salamanders and frogs) whose populations mainly differentiate via isolation-by-distance (IBD) and allopatric speciation has been the primary mode responsible for species diversification because of their low vagility and strong niche conservatism in fragmented landscapes (Kozak et al.^2006^; Fouquet et al.^2007^). As such, a positive correlation between genetic and geographic distances is expected among populations of a species and IBD might still be observed among closely related and recently diversified species (Fouquet et al.^2007^), resulting in geographically constrained monophyly in species (Fouquet et al.^2007^; Hughes and Hollingsworth^2008^; Dewitte et al.^2011^). The predicted phylogenetic and population genetic patterns thus form testable hypotheses for further population genetic studies and better sampled *Begonia* phylogenetic analyses as well as other limestone cave plants in the Sino-Vietnamese limestone karst region and other major karst ecosystems worldwide.

### Taxonomic Treatment

Synopsis of *Begonia* sect. *Coelocentrum* Irmsch.

***Begonia*** **sect.** ***Coelocentrum*** Irmsch., Mitt. Inst. Allg. Bot. Hamburg 10: 533. 1939; Doorenbos et al., The section of *Begonia* 84. 1998; Ku, Fl. Reipubl. Popularis Sin. 52(1): 127, 129. 1999; Shui et al., Bot. Bull. Acad. Sin. 43: 314. 2002; Gu, Fl. China 13: 205. 2007. LECTOTYPE SPECIES: *B. porteri* H. Lév. & Vaniot (designated by Barkley and Baranov^1972^).—*Begonia* sect. *Leprosae* (T.C. Ku) Y.M. Shui, Bot. Bull. Acad. Sin. 43: 321. 2002, syn. nov.

Herbs monoecious, terrestrial, perennial, rhizomatous without erect stems and tubers, acaulescent, rhizome herbaceous or slightly woody. Leaves alternate, variable, mostly broadly ovate, oblique at base, rarely palmate, palmately compound, or occasionally peltate, glabrous to tomentose, often variegated. Inflorescence axillary, cymose (dichasial or dichasial at base and monochasial at apex), rarely thyrsoid, staminate flowers basal and carpellate flowers distal, protandrous. Perianth segments free, usually white or pink, rarely greenish or yellowish. Staminate flower with 4, or rarely 2 tepals. Carpellate flowers usually with 3 tepals, rarely 2 or 5 (–6); ovary 3-winged, or rarely unwinged, wings equal to unequal in fruit, mostly 1-locular, parietal placentation, rarely 3-locular with axile placentation. Fruit a 3-winged capsule, nodding, or rarely berry-like, cylindric.

*Cytology*. 2*n* = 30 or rarely 45 (Peng et al.[Bibr CR47][Bibr CR48][Bibr CR50][Bibr CR51][Bibr CR52][Bibr CR53];Gu et al. 2007; Liu et al.^2007^).

*Distribution* and *Habitat*. Currently 61 species, two varieties, and one natural hybrid distributed in the caves, fissures and forest floors of limestone karsts in southern China (Guangxi, southern Guizhou, and southeastern Yunnan provinces) and northern Vietnam, at elevations between ca. 100 and 1,300 m.

*Species Included and Distribution—B. arachnoidea* C.I Peng, S.M. Ku & Yan Liu (China: Guangxi), *B. asteropyrifolia* Y.M. Shui & W.H. Chen (China: Guangxi), *B. aurantiflora* C.I Peng, Yan Liu & S.M. Ku (China: Guangxi), *B. auritistipula* Y.M. Shui & W.H. Chen (China: Guangxi), *B. austroguangxiensis* Y.M. Shui & W.H. Chen (China: Guangxi), *B. babeana* Aver. & H.Q. Nguyen (Vietnam), *B. bamaensis* Yan Liu & C.I Peng (China: Guangxi), *B. biflora* T.C. Ku (China: Yunnan; Vietnam), *B.* ×*breviscapa* C.I Peng, Yan Liu & S.M. Ku (China: Guangxi), *B. cavaleriei* H. Lév. (China: Guangxi, Yunnan, Guizhou; Vietnam), *B. chongzuoensis* Yan Liu, S.M. Ku & C.I Peng (China: Guangxi), *B. cirrosa* L.B. Sm. & Wassh. (China: Guangxi, Yunnan), *B. crystallina* Y.M. Shui & W.H. Chen (China: Yunnan), *B. curvicarpa* S.M. Ku, C.I Peng & Yan Liu (China: Guangxi), *B. cylindrica* D.R. Liang & X.X. Chen (China: Guangxi), *B. daxinensis* T.C. Ku (China: Guangxi), *B. debaoensis* C.I Peng, Yan Liu & S.M. Ku (China: Guangxi), *B. fangii* Y.M. Shui & C.I Peng (China: Guangxi), *B. ferox* C.I Peng & Yan Liu (China: Guangxi), *B. filiformis* Irmsch. (China: Guangxi), *B. fimbribracteata* Y.M. Shui & W.H. Chen (China: Guangxi), *B. guangxiensis* C.Y. Wu (China: Guangxi), *B. huangii* Y.M. Shui & W.H. Chen (China: Yunnan), *B. jingxiensis* D. Fang & Y.G. Wei (China: Guangxi), *B. kui* C.I Peng (Vietname), *B. lanternaria* Irmsch. (China: Guangxi; Vietnam), *B. leprosa* Hance (China: Guangxi, Guangdong), *B. liuyanii* C.I Peng, S.M. Ku & W.C. Leong (China: Guangxi), *B. longgangensis* C.I Peng & Yan Liu (China: Guangxi), *B. longistyla* Y.M. Shui & W.H. Chen (China: Yunnan), *B. luochengensis* S.M. Ku, C.I Peng & Yan Liu (China: Guangxi), *B. luzhaiensis* T.C. Ku (China: Guangxi), *B. mashanica* D. Fang & D.H. Qin (China: Guangxi), *B. masoniana* Irmsch. (China: Guangxi; Vietnam), *B. morsei* Irmsch. (China: Guangxi), *B. nahangensis* Aver. & H.Q. Nguyen (Vietnam), *B. ningmingensis* D. Fang, Y.G. Wei & C.I Peng (China: Guangxi), *B. ningmingensis* var. *bella* D. Fang, Y.G. Wei & C.I Peng (China: Guangxi), *B. obliquifolia* S.H. Huang & Y.M. Shui (China: Yunnan), *B. ornithophylla* Irmsch. (China: Guangxi), *B. pengii* S.M. Ku & Yan Liu (China: Guangxi), *B. phuthoensis* H.Q. Nguyen (Vietnam), *B. picturata* Yan Liu, S.M. Ku & C.I Peng (China: Guangxi), *B. platycarpa* Y.M. Shui & W.H. Chen (China: Yunnan), *B. porteri* H. Lév. & Vaniot (China: Guangxi, Guizhou), *B. porteri* var. *macrorhiza* Gagnep. (Vietnam), *B. pseudodaxinensis* S.M. Ku, Yan Liu & C.I Peng (China: Guangxi), *B. pseudodryadis* C.Y. Wu (China: Yunnan), *B. pseudoleprosa* C.I Peng, Yan Liu & S.M. Ku (China: Guangxi), *B. pulvinifera* C.I Peng & Yan Liu (China: Guangxi), *B. retinervia* D. Fang, D.H. Qin & C.I Peng (China: Guangxi), *B. rhynchocarpa* Y.M. Shui & W.H. Chen (China: Yunnan), *B. rugosula* Aver. (Vietnam), *B. semiparietalis* Yan Liu, S.M. Ku & C.I Peng (China: Guangxi), *B. setulosopeltata* C.Y. Wu (China: Guangxi), *B. sinofloribunda* Dorr (China: Guangxi), *B. sonlaensis* Aver. (Vietnam), *B. subcoriacea* C.I Peng, Yan Liu & S.M. Ku (China: Guangxi), *B. suboblata* D. Fang & D.H. Qin (China: Guangxi), *B. umbraculifolia* Y. Wan & B.N. Chang (China: Gaungxi), *B. variifolia* Y.M. Shui & W.H. Chen (China: Guangxi), *B. wangii* T.T. Yu (China: Guangxi, Yunnan), *B. zhengyiana* Y.M. Shui (China: Yunnan).

Notes—Unpublished data in our ongoing phylogenetic study indicates that *B. wangii* T.T. Yu, previously assigned to sect. *Diploclinium* (Shui et al.^2002^), is nested firmly within Clade *SVLB* and hereby transferred to sect. *Coelocentrum*.

## Electronic supplementary material

Additional file 1:***Voucher information and GenBank accession numbers.***(PDF 158 KB)

Below are the links to the authors’ original submitted files for images.

Authors’ original file for figure 1

Authors’ original file for figure 2
