# Supplementary material for: Phylogenetic analyses of Begonia sect. Coelocentrum and allied limestone species of China shed light on the evolution of Sino-Vietnamese karst flora
Source: Bot Stud. 2014 Jan 7;55:1. doi: 10.1186/1999-3110-55-1 (PMC5432845; doi:10.1186/1999-3110-55-1)
Supplement: Supplementary file 1 — Additional file 1:Voucher information and GenBank accession numbers.(PDF 158 KB) [file 40529_2013_53_MOESM1_ESM.pdf]

Additional file 1. Voucher information and GenBank accession numbers.

| Taxon name | Section (Abbreviation) | Country (Region) | Voucher specimen | *rpL16* | ITS |
|---|---|---|---|---|---|
| ***Begonia aequata*** A. Gray | *Petermannia* (PET)[1] | Philippines | *Peng 19915* | KF707931 | AF485147 |
| ***Begonia alicida*** C.B. Clarke ex Hook. f. | *Alicida* (ALI)[1] | Thailand | *Peng 21033* | KF707932 | KF636419 |
| ***Begonia alveolata*** T.T. Yu | *Diploclinium* (DIP)[9] | China (Yunnan) | *Peng 20421* | KF707933 | AY048977 |
| ***Begonia amphioxus*** Sands | *Petermannia* (PET)[3] | Malaysia | *Peng 21212* | KF707934 | AF485150 |
| ***Begonia arachnoidea*** C.I Peng, Yan Liu & S.M. Ku | *Coelocentrum* (COE)[6] | China (Guangxi) | *Peng 19762* | KF707935 | KF636420 |
| ***Begonia augustinei*** Hemsl. | *Platycentrum* (PLA)[2] | China (Yunnan) | *Peng 20759* | KF707936 | KF636421 |
| ***Begonia auritistipula*** Y.M. Shui & W.H. Chen | *Coelocentrum* (COE)[2] | China (Yunnan) | *Peng 20532* | KF707937 | KF636422 |
| ***Begonia austroguangxiensis*** Y.M. Shui & W.H.Chen | *Coelocentrum* (COE)[2] | China (Guangxi) | *Peng 19788* | KF707938 | KF636423 |
| ***Begonia balansana*** Gagnep. | *Sphenanthera* (SPH)[9] | Vietnam | *Peng 21928* | KF707939 | AF485091 |
| ***Begonia bamaensis*** Yan Liu & C.I Peng | *Coelocentrum* (COE)[5] | China (Guangxi) | *Peng 18752* | KF707940 | KF636424 |
| ***Begonia bataiensis*** Kiew | *Leprosae* (LEP)[3] | Vietnam | *Peng 20264* | KF707941 | KF636425 |
| ***Begonia berhamanii*** Kiew | *Petermannia* (PET)[3] | Malaysia (Sabah) | *Peng 21974* | KF707942 | KF636426 |
| ***Begonia bipinnatifida*** J.J. Sm. | *Petermannia* (PET)[3] | Papua New Guinea | *Peng s.n.* | KF707943 | KF636427 |
| ***Begonia boisiana*** Gagnep. | *Ignota*(UA)[3] | Vietnam | *Peng 21416* | KF707944 | AF534719 |
| ***Begonia bolsteri*** Merr. | *Petermannia* (PET)[3] | Philippines | *Rubite 320* | KF707945 | KF636428 |
| ***Begonia brevipes*** Merr. | *Petermannia* (PET)[3] | Philippines (Luzon) | *Rubite 113* | KF707946 | KF636429 |
| ***Begonia brevirimosa*** Irmsch. | *Petermannia* (PET)[3] | Papua New Guinea | *Peng 20235-b* | KF707947 | AF485145 |
| ***Begonia cathayana*** Hemsl. | *Platycentrum* (PLA)[9] | China (Guangxi) | *Peng 20288* | KF707948 | AF280106 |
| ***Begonia cavaleriei*** H. Lév. | *Diploclinium* (DIP)[9] | Vietnam | *Peng 19933* | KF707949 | KF636430 |
| ***Begonia*** cf. ***anthonyi*** Kiew | *Petermannia* (PET)[3] | Malaysia (Sabah) | *Peng 21963* | KF707950 | KF636431 |
| ***Begonia chingii*** Irmsch. | *Reichenheimia* (REI)[9] | China (Guangxi) | *Peng 19733* | KF707951 | KF636432 |
| ***Begonia chloroneura*** P. Wilkie & Sands | *Baryandra* (BAR)[7] | Philippines | *Peng 21217* | KF707952 | AF485134 |
| ***Begonia cirrosa*** L.B. Sm. & Wassh. | *Coelocentrum* (COE)[9] | China (Yunnan) | *Peng 20518* | KF707953 | AY048979 |
| ***Begonia contracta*** Warb. | *Petermannia* (PET)[3] | Philippines (Luzon) | *Rubite 120* | KF707954 | KF636433 |
| ***Begonia cylindrica*** D.R. Liang & X.X. Chen | *Leprosae* (LEP)[9] | China (Guangxi) | *Leong 3636* | KF707955 | KF636434 |
| ***Begonia decora*** Stapf | *Platycentrum* (PLA)[3] | Malaysia | *Peng 20261* | KF707956 | KF636435 |
| ***Begonia dipetala*** Graham | *Haagea* (HAA)[1] | India | *Peng 21265* | KF707957 | AF469124 |
| ***Begonia dregei*** Otto & A. Dietr. | *Augustia* (AUG)[1] | South Africa | *Peng s.n.* | KF707958 | AY429336 |
| ***Begonia dryadis*** Irmsch. | *Platycentrum* (PLA)[9] | China (Yunnan) | *Peng 18016* | KF707959 | KF636436 |
| ***Begonia edulis*** H. Lév. | *Platycentrum* (PLA)[9] | China (Guangxi) | *Peng 18747* | KF707960 | KF636437 |
| ***Begonia erythrogyna*** Sands | *Petermannia* (PET)[9] | Malaysia (Sabah) | *Peng 21978* | KF707961 | KF636438 |
| ***Begonia fimbristipula*** Hance | *Diploclinium* (DIP)[9] | China (Guangxi) | *Leong 3673* | KF707962 | KF636439 |
| ***Begonia fuscisetosa*** Sands | *Petermannia* (PET)[3] | Malaysia (Sabah) | *Peng 22013* | KF707963 | KF636440 |
| ***Begonia goegoensis*** N.E. Br. | *Reichenheimia* (REI)[3] | Malaysia | *Peng 20238* | KF707964 | AF485138 |
| ***Begonia grandis*** subsp. ***holostyla*** Irmsch. | *Diploclinium* (DIP)[9] | China (Guizhou) | *Peng 18817* | KF707965 | AF485088 |
| ***Begonia gueritziana*** Gibbs | *Baryandra* (BAR)[7] | Malaysia (Sabah) | *Peng 21976* | KF707966 | KF636441 |
| ***Begonia guixiensis*** sp. ined. | *Coelocentrum* (COE) | China (Guangxi) | *Peng 20310* | KF707967 | KF636442 |
| ***Begonia hainanensis*** Chun & F. Chun | *Petermannia* (PET)[2, 8] | China (Hainan) | *Peng 19543* | KF707968 | KF636443 |
| ***Begonia handelii*** Irmsch. | *Sphenanthera* (SPH)[9] | China (Yunnan) | *Peng 17513* | KF707969 | AY048982 |
| ***Begonia hatacoa*** Buch.-Ham. ex D. Don | *Platycentrum* (PLA)[9] | China | *Peng 20861* | KF707970 | KF636444 |

| | | | | | |
|---|---|---|---|---|---|
| ***Begonia hemsleyana*** Hook. f. | *Platycentrum* (PLA)[9] | China (Yunnan) | *Peng 17590* | KF707971 | AF485099 |
| ***Begonia hernandioides*** Merr. | *Baryandra* (BAR)[3] | Philippines (Luzon) | *Rubite 106* | KF707972 | KF636445 |
| ***Begonia inostegia*** Stapf | *Petermannia* (PET)[3] | Malaysia (Sabah) | *Peng 22023* | KF707973 | KF636446 |
| ***Begonia isoptera*** Dryand. ex Sm. | *Petermannia* (PET)[3] | Indonesia (Java) | *Peng 21301* | KF707974 | KF636447 |
| ***Begonia jingxiensis*** D. Fang & Y.G. Wei | *Coelocentrum* (COE)[9] | China (Guangxi) | *Peng 19742* | KF707975 | KF636448 |
| ***Begonia mashanica*** D. Fang & D.H. Qin | *Coelocentrum* (COE)[9] | China (Guangxi) | *Peng 19805* | KF707976 | KF636449 |
| ***Begonia kinabaluensis*** Sands | *Petermannia* (PET)[3] | Malaysia (Sabah) | *Peng 22010* | KF707977 | KF636450 |
| ***Begonia kingiana*** Irmsch. | *Ridleyella* (RID)[3] | Malaysia | *Peng 21226* | KF707978 | KF636451 |
| ***Begonia labordei*** H. Lév. | *Diploclinium* (DIP)[9] | China (Yunnan) | *Peng 20520* | KF707979 | KF636452 |
| ***Begonia lagunensis*** Elmer | *Petermannia* (PET)[3] | Philippines (Luzon) | *Rubite 285* | KF707980 | KF636453 |
| ***Begonia lambii*** Kiew | *Petermannia* (PET)[3] | Malaysia (Sabah) | *Peng 21966* | KF707981 | KF636454 |
| ***Begonia laminariae*** Irmsch. | *Platycentrum* (PLA)[9] | China (Yunnan) | *Peng 17447* | KF707982 | KF636455 |
| ***Begonia lanternaria*** Irmsch. | *Coelocentrum* (COE)[9] | China (Guangxi) | *Peng 19789* | KF707983 | KF636456 |
| ***Begonia leprosa*** Hance | *Leprosae* (LEP)[9] | China (Guangdong) | *Peng 19474* | KF707984 | KF636457 |
| ***Begonia liuyanii*** C.I Peng, S.M. Ku & W.C. Leong | *Coelocentrum* (COE)[9] | China (Guangxi) | *Peng 18820* | KF707985 | KF636458 |
| ***Begonia longa*** sp. ined. | *Coelocentrum* (COE) | Vietnam | *Peng 20073* | KF707986 | KF636459 |
| ***Begonia longicarpa*** K.Y. Guan & D.K. Tian | *Leprosae* (LEP)[9] | China (Yunnan) | *Peng 18651* | KF707987 | AY048985 |
| ***Begonia longifolia*** Blume | *Sphenanthera* (SPH)[9] | Taiwan | *Peng 16795* | KF707988 | AF485105 |
| ***Begonia longistyla*** Y.M. Shui & W.H. Chen | *Coelocentrum* (COE)[2] | China (Yunnan) | *Peng 20435* | KF707989 | KF636460 |
| ***Begonia luzhaiensis*** T.C. Ku | *Coelocentrum* (COE)[9] | China (Guangxi) | *Ku 2019* | KF707990 | KF636461 |
| ***Begonia madaiensis*** Kiew | *Petermannia* (PET)[3] | Malaysia | *Peng 21981* | KF707991 | KF636462 |
| ***Begonia masoniana*** Irmsch. ex Ziesenh. | *Coelocentrum* (COE)[9] | China (Guangxi) | *Peng 19795* | KF707992 | KF636463 |
| ***Begonia merrittii*** Merr. | *Petermannia* (PET)[3] | Philippines (Luzon) | *Rubite 137* | KF707993 | KF636464 |
| ***Begonia nigritarum*** (Kamel) Steud. | *Baryandra* (BAR)[3] | Philippines | *Rubite 346* | KF707994 | KF636465 |
| ***Begonia ningmingensis*** D. Fang, Y.G. Wei & C.I Peng | *Coelocentrum* (COE)[2] | China (Guangxi) | *Leong 3410* | KF707995 | KF636466 |
| ***Begonia oreodoxa*** Chun & F. Chun ex G.Y. Wu & T.C. Ku | *Platycentrum* (PLA)[9] | China (Yunnan) | *Peng 20454* | KF707996 | KF636467 |
| ***Begonia oxysperma*** A. DC. | *Baryandra* (BAR)[7] | Philippines (Luzon) | *Rubite 213* | KF707997 | AF485131 |
| ***Begonia palmata*** D. Don | *Platycentrum* (PLA)[9] | Taiwan | *Peng 20993* | KF707998 | KF636468 |
| ***Begonia panayensis*** Merr. | *Petermannia* (PET)[3] | Philippines (Panay) | *Rubite 328* | KF707999 | KF636469 |
| ***Begonia paracauliflora*** sp. ined. | *Petermannia* (PET)[3] | Malaysia (Sabah) | *Peng 21964* | KF708000 | KF636470 |
| ***Begonia parvula*** H. Lév. & Vaniot | *Reichenheimia* (REI)[9] | China (Yunnan) | *Peng 20396* | KF708001 | KF636471 |
| ***Begonia pavonina*** Ridl. | *Platycentrum* (PLA)[3] | Malaysia | *Peng 20239* | KF708002 | KF636472 |
| ***Begonia pedatifida*** H. Lév. | *Platycentrum* (PLA)[9] | China (Guizhou) | *Peng 18779* | KF708003 | KF636473 |
| ***Begonia peltatifolia*** H.L. Li | *Diploclinium* (DIP)[9] | China (Hainan) | *Peng 19504* | KF708004 | KF636474 |
| ***Begonia pengii*** S.M. Ku & Yan Liu | *Coelocentrum* (COE)[4] | China (Guangxi) | *Peng 21070* | KF708005 | KF636475 |
| ***Begonia pseudolateralis*** Warb. | *Petermannia* (PET)[3] | Philippines (Luzon) | *Rubite 490* | KF708006 | KF636476 |
| ***Begonia pulvinifera*** C.I Peng & Yan Liu | *Diploclinium* (DIP)[2] | China (Guangxi) | *Peng 19741* | KF708007 | KF636477 |
| ***Begonia ramosii*** Merr. | *Petermannia* (PET)[3] | Philippines | *Rubite 305* | KF708008 | KF636478 |
| ***Begonia ravenii*** C.I Peng & Y.K. Chen | *Diploclinium* (DIP)[9] | Taiwan | *Peng 14855* | KF708009 | KF636479 |
| ***Begonia retinervia*** D. Fang, D.H. Qin & C.I Peng | *Coelocentrum* (COE)[2] | China (Guangxi) | *Peng 19808* | KF708010 | KF636480 |
| ***Begonia ruboides*** C.M. Hu ex C.Y. Wu & T.C. Ku | *Diploclinium* (DIP)[9] | China (Yunnan) | *Peng 18705* | KF708011 | KF636481 |
| ***Begonia rufipila*** Merr. | *Baryandra* (BAR)[7] | Philippines (Luzon) | *Rubite 265* | KF708012 | KF636482 |

| | | | | | |
|---|---|---|---|---|---|
| ***Begonia semiparietalis*** Y. Liu, S.M. Ku & C.I Peng | *Coelocentrum* (COE)[2] | China (Guangxi) | *Peng 20327* | KF708013 | KF636483 |
| ***Begonia serratipetala*** Irmsch. | *Petermannia* (PET)[3] | Papua New Guinea | *Peng 21222* | KF708014 | KF636484 |
| ***Begonia sikkimensis*** A. DC. | *Platycentrum* (PLA)[9] | China (Xizang) | *Peng 20848* | KF708015 | KF636485 |
| ***Begonia sinofloribunda*** Dorr | *Petermannia* (PET)[8] | China (Guangxi) | *Leong 3401* | KF708016 | KF636486 |
| ***Begonia subnummularifolia*** Merr. | *Baryandra* (BAR)[7] | Malaysia (Sabah) | *Peng s.n.* | KF708017 | KF636487 |
| ***Begonia sutherlandii*** Hook. f. | *Augustia* (AUG)[1] | South Africa | *Jager 1200-5* | KF708018 | AF485215 |
| ***Begonia symsanguinea*** L. Forrest & Hollingsw. | *Symbegonia* (SYM)[3] | Papua New Guinea | *Peng 21204* | KF708019 | AF485151 |
| ***Begonia taiwaniana*** Hayata | *Diploclinium* (DIP)[9] | Taiwan | *Peng 18111* | KF708020 | KF636488 |
| ***Begonia variabilis*** Ridl. | *Parvibegonia* (PAR)[1] | Thailand | *Peng 21040* | KF708021 | AY753732 |
| ***Begonia variegata*** Y. M. Shui & W. H. Chen | *Coelocentrum* (COE)[2] | China (Yunnan) | *Peng 20533* | KF708022 | KF636489 |
| ***Begonia versicolor*** Irmsch. | *Platycentrum* (PLA)[9] | China (Yunnan) | *Peng 18688* | KF708023 | AF485090 |
| ***Begonia wadei*** Merr. & Quisumb. | *Baryandra* (BAR)[7] | Philippines | *Rubite 356* | KF708024 | KF636490 |
| ***Begonia wenshanensis*** C.M. Hu ex C.Y. Wu & T.C. Ku | *Diploclinium* (DIP)[9] | China (Yunnan) | *Peng 20516* | KF708025 | AY048974 |
| ***Begonia yappii*** Ridl. | *Diploclinium* (DIP)[1] | Malaysia | *Peng 20243* | KF708026 | KF636491 |

## References


[1]Doorenbos J, Sosef MSM, Wilde JJFEd (1998) The sections of *Begonia*. Wageningen Agriculture University, Wageningen

[2]Gu C-Z (2007) Infrageneric classification of *Begonia*. In: Wu Z-Y, Raven PH, Hong D-Y (eds) Flora of China, vol. 13. Science Press and Missouri Botanical Garden Press, Beijing and St. Louis, pp 205–207

[3]Hughes M, Pullan M (2007) Southeast Asian *Begonia* Database. http://www.rbge.org.uk

[4]Ku S-M, Kono Y, Liu Y (2008) *Begonia pengii* (sect. *Coelocentrum*, Begoniaceae), a new species from limestone areas in Guangxi, China. Bot Stud 49:167–175

[5]Liu Y, Ku S-M, Peng C-I (2007) *Begonia bamaensis* (sect. *Coelocentrum*, Begoniaceae), a new species from limestone areas in Guangxi, China. Bot Stud 48:465–473

[6]Peng C-I, Ku S-M, Kono Y, Chung K-F, Liu Y (2008a) Two new species of *Begonia* (sect. *Coelocentrum*, Begoniaceae) from limestone areas in Guangxi, China: *B. arachnoidea* and *B. subcoriacea*. Bot Stud 49:405–418

[7]Rubite RR, Hughes M, Alejandro GJD, Peng C-I (2013) Recircumscription of *Begonia* sect. *Baryandra* (Begoniaceae): evidence from molecular data. Bot Stud 54:38

[8]Shui Y-M, Chen W-H (2004) Revision to sect. *Petermannia* of *Begonia* (Begoniaceae) in China. Acta Bot Yunnanica 26:482–486

[9]Shui Y-M, Peng C-I, Wu C-Y (2002) Synopsis of the Chinese species of *Begonia* (Begoniaceae), with a reappraisal of sectional delimitation. Bot Bull Acad Sin 43:313–327
