# Supplementary figures and images for: Phylogenetic analyses of Begonia sect. Coelocentrum and allied limestone species of China shed light on the evolution of Sino-Vietnamese karst flora
Source: Bot Stud. 2014 Jan 7;55:1. doi: 10.1186/1999-3110-55-1 (PMC5432845; doi:10.1186/1999-3110-55-1)

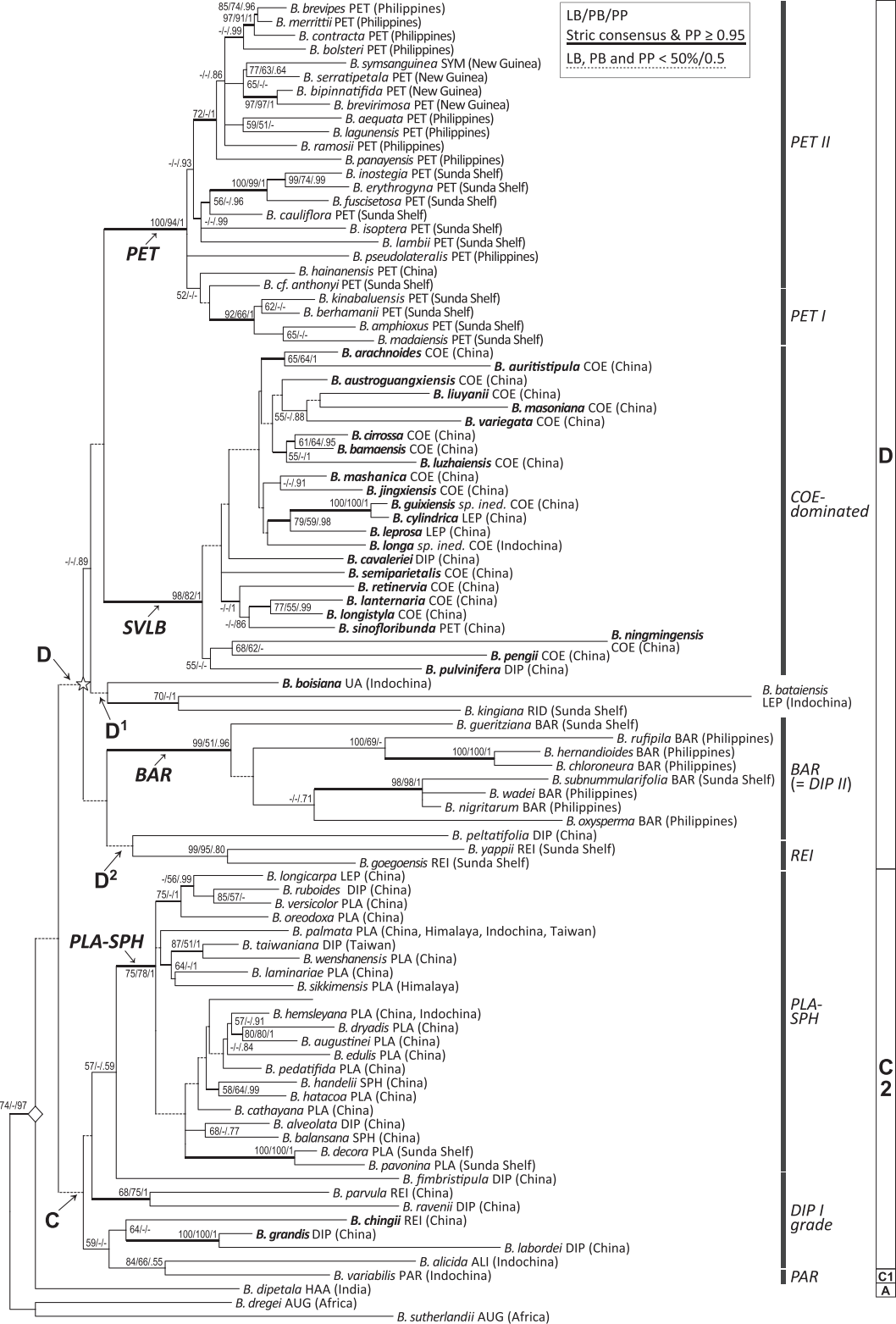

Supplement: Supplementary file 2 — Authors’ original file for figure 1 [file 40529_2013_53_MOESM2_ESM.pdf]

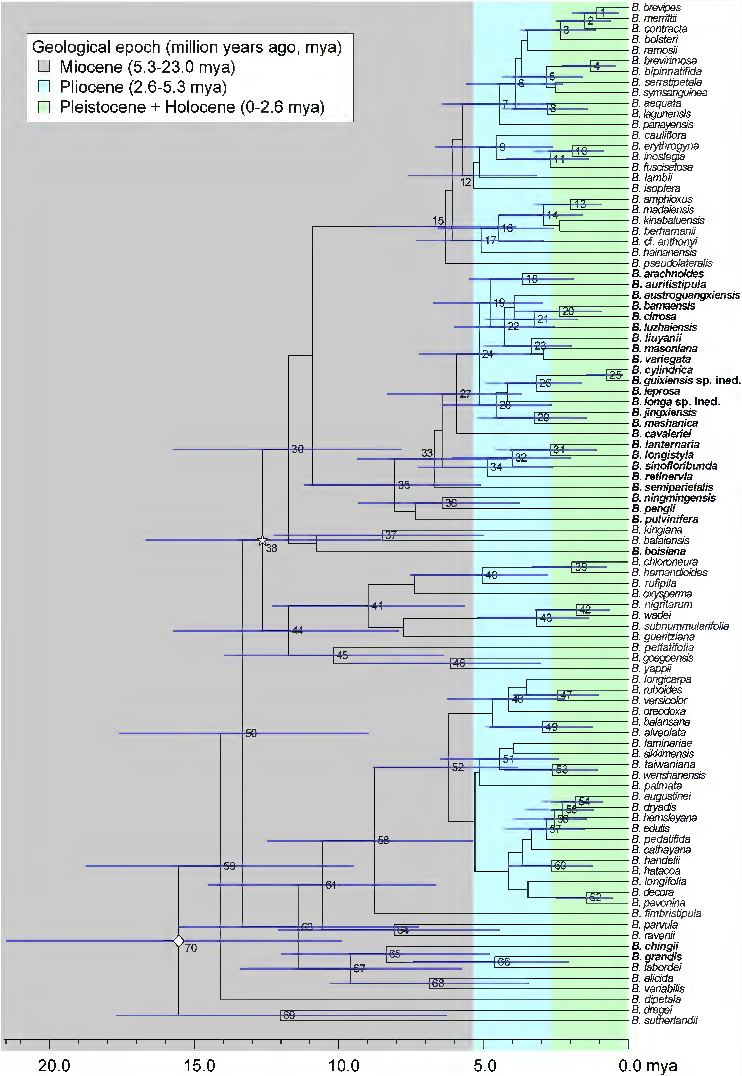

Supplement: Supplementary file 3 — Authors’ original file for figure 2 [file 40529_2013_53_MOESM3_ESM.tiff]
